# Effect of tyrosine autophosphorylation on catalytic activity and subcellular localisation of homeodomain-interacting protein kinases (HIPK)

**DOI:** 10.1186/s12964-014-0082-6

**Published:** 2015-01-29

**Authors:** Jan van der Laden, Ulf Soppa, Walter Becker

**Affiliations:** Institute of Pharmacology and Toxicology, RWTH Aachen University, Wendlingweg 2, 52057 Aachen, Germany

**Keywords:** Protein kinase, Dual specificity, Tyrosine autophosphorylation, Activation loop, Subcellular localisation, p27^Kip1^, HIPK, DYRK

## Abstract

**Background:**

Homeodomain interacting protein kinases (HIPKs) function as modulators of cellular stress responses and regulate cell differentiation, proliferation and apoptosis. The HIPK family includes HIPK1, HIPK2 and HIPK3, which share a similar domain structure, and the more distantly related HIPK4. Although HIPKs phosphorylate their substrates on serine or threonine residues, it was recently reported that HIPK2 depends on the autophosphorylation of a conserved tyrosine in the activation loop to acquire full catalytic activity and correct subcellular localization. In this study we addressed the question whether tyrosine autophosphorylation in the activation loop has a similar function in the other members of the HIPK family.

**Results:**

All HIPKs contained phosphotyrosine when expressed in HeLa cells. Catalytically inactive point mutants were not tyrosine-phosphorylated, indicating that HIPKs are dual-specificity protein kinases that autophosphorylate on tyrosine residues. HIPK point mutants lacking the conserved tyrosine residue in the activation loop showed reduced catalytic activity towards peptide and protein substrates. Analysis of these mutants revealed that HIPK1, HIPK2 and HIPK3 but not HIPK4 are capable of autophosphorylating on other tyrosines. Inhibition of tyrosine phosphatase activity by treatment with vanadate enhanced global phosphotyrosine content of HIPK1, HIPK2 and HIPK3 but did not affect tyrosine phosphorylation in the activation loop. Mutation of the activation-loop tyrosines resulted in a redistribution of HIPK1 and HIPK2 from a speckle-like subnuclear compartment to the cytoplasm, whereas catalytically inactive point mutants showed the same pattern of cellular distribution as the wild type proteins. In contrast, mutation of the activating tyrosine did not increase the low percentage of cells with extranuclear HIPK3. HIPK4 was excluded from the nucleus with no difference between the wild type kinase and the point mutants.

**Conclusions:**

These results show that HIPKs share the mechanism of activation by tyrosine autophosphorylation with the closely related DYRK family (dual-specificity tyrosine phosphorylation regulated kinase). However, members of the HIPK family differ regarding the subcellular localization and its dependence on tyrosine autophosphorylation.

**Electronic supplementary material:**

The online version of this article (doi:10.1186/s12964-014-0082-6) contains supplementary material, which is available to authorized users.

## Background

Homeodomain-interacting protein kinases (HIPK1, HIPK2, HIPK3) were originally identified as proteins that interact with the homeobox transcription factors NKx-1.2 [1]. HIPK1-3 share about 90% identical amino acids in their kinase domains and are also related in the architecture of the noncatalytic regions. These include a smaller N-terminal part with a conserved sumoylation site and a large C-terminal domain that harbours a homeobox-interaction domain, an autoinhibitory domain, a region rich in proline, glutamic acid, serine and threonine residues (PEST), and a C-terminus rich in short repeats of serines, glutamines and alanines (SQA region, also known as tyrosine/histidine (YH)-rich region) [1,2]. HIPK4 was later identified by its sequence similarity with HIPK1, 2 and 3 and is the most divergent member of the HIPK family [3,4]. HIPK4 is not structurally related with HIPK1-3 outside the catalytic domain [4].

HIPK2, the best-studied member of the HIPK family, is involved in the regulation of various cellular processes including differentiation, proliferation, apoptosis and stress response to DNA damage and hypoxia [5,6]. Perhaps most importantly, HIPK2 promotes apoptosis upon genotoxic stress by phosphorylating the tumor suppressor protein p53 at Ser46 [7-10]. Obviously, the function of HIPK2 as a regulator of cellular life and death must itself be tightly controlled. Accordingly, HIPK2 activity is regulated by several mechanism including degradative ubiquitination, caspase-mediated cleavage of an autoinhibitory domain, sumoylation, acetylation and phosphorylation [11,12]. Most effects of HIPK2 are mediated by transcriptional regulation, as HIPK2 can either activate or repress gene expression by interaction with components of the transcription machinery [13-15].

Whereas more and more putative targets of HIPK2 are being identified [15], enhancing our understanding of its function in cell death and cell survival, much less is known about the other members of the HIPK family. Due to their highly similar primary structures, HIPK1 and HIPK2 are assumed to have at least a certain degree of redundant activity [16]. Whereas HIPK1/2 double knockout mice die at embryonic day 12.5, either HIPK1 or HIPK2-deficient mice develop grossly normal but exhibit differences in apoptosis induction and eye development [17-20]. Although the cellular functions of HIPK1 are not very well defined, available evidence indicates a role in the regulation of apoptosis by interaction with nuclear proteins [16,21]. HIPK3 has been characterized as a regulator of the androgen receptor and Runt-related transcription factor 2 (Runx2) [22,23]. Recently, HIPK3^−/−^ mice were shown to have impaired glucose-induced insulin secretion [24], and HIPK3 has been implicated in the pathogenesis of human type 2 diabetes [25]. Compared to HIPK2, HIPK1 and HIPK3 are less well characterized regarding their molecular and cellular function, and it is unclear to which degree these kinases fulfill redundant or divergent tasks. Almost nothing is known about HIPK4 except for its capacity to phosphorylate p53 at Ser9 [4].

Consistent with their roles in transcriptional regulation, HIPK1, HIPK2 and HIPK3 are predominantly nuclear proteins that are concentrated in subnuclear structures that appear as a punctate pattern [1,21,22,26]. These speckles are distinct from other subnuclear structures and were thus designated HIPK domains [9]. Interestingly, the punctate nuclear distribution of HIPK2 depends on a functional SUMO interaction motif that is also present in HIPK1 and HIPK3 [27]. However, in some cells HIPK2 is localised diffusely in the nucleus and also in the cytoplasm [9]. These findings suggest that the subcellular distribution of HIPK2, and possibly of HIPK1 and HIPK3, is a regulated process relevant for the biological function of these proteins. In contrast to HIPK1-3, HIPK4 lacks an apparent nuclear localization signal and was found to be evenly distributed in the cytoplasm [4].

Although HIPKs phosphorylate substrates on serine or threonine residues, HIPK2 has recently been shown to autophosphorylate at a specific, highly conserved tyrosine residue following the Mg^2+^-binding DFG motif in the activation loop of the catalytic domain [28,29]. This tyrosine is not only present in all HIPKs but also in other kinases of the CMGC group including the MAPK family, GSK3 and the closely related DYRKs [30]. In the MAPK family, the corresponding tyrosine belongs to a Thr-x-Tyr motif. Dual phosphorylation of this motif by specific upstream kinases serves as a regulatory switch that controls catalytic activity. In contrast, DYRKs and GSK3 autophosphorylate the activation loop-tyrosine already during translation, which induces the constitutive activity of these kinases [31-34]. Similar to the DYRKs, tyrosine autophosphorylation of HIPK2 is necessary for full catalytic activity of the mature kinase [28,29]. We and others have recently shown that DYRK1A and HIPK2 are not only capable of co-translationally phosphorylating the crucial tyrosine in the activation loop, but can also as mature kinases autophosphorylate on tyrosines outside the catalytic domain [29,34]. In contrast to DYRKs, tyrosine phosphorylation of HIPK2 has been reported to be at least partially under the control of upstream kinases [35,36]. Specifically, HIPK2 has been shown to be phosphorylated by the tyrosine kinase Src on numerous residues, including Y361 [36]. Phosphoproteomics screens have provided ample evidence that the activation loop tyrosine is also phosphorylated in HIPK1, HIPK3 and HIPK4 (phosphosite.org). However, it is unknown whether these kinases also autophosphorylate on tyrosine and whether tyrosine phosphorylation is functionally important. The present study aims at a comparative analysis of the role of the activation loop tyrosine in all members of the HIPK family.

## Results and discussion

### Tyrosine phosphorylation of HIPKs

**Figure 1:**
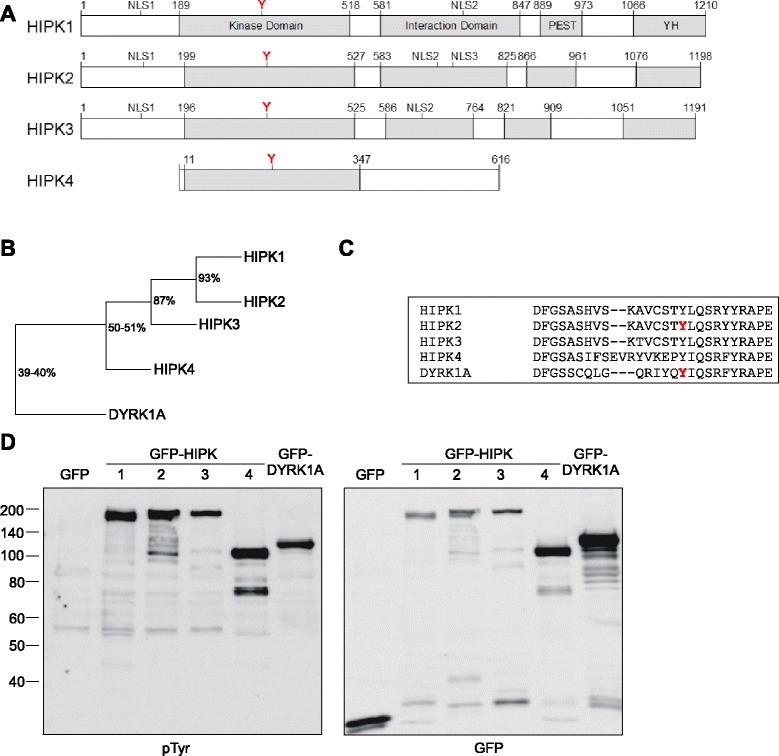
**Comparison of HIPK structures and tyrosine phosphorylation. A**, Schematic illustration of HIPK1-4. The definition of the domains in HIPK1-3 was adapted from Kim et al. [26]. Nuclear localisation sequences (NLS) and the tyrosine residue in the activation loop (Y) are indicated. **B**, The tree illustrates the sequence identity of the catalytic domains of HIPK1-4 and DYRK1A and panel **C** shows the alignment of the activation loop sequences. Tyrosine residues known to be autophosphorylated in HIPK2 and DYRK1A are highlighted (red) [28,29,34]. **D**, GFP fusion proteins of HIPK1-4 and DYRK1A were immunoprecipitated from transiently transfected HeLa cells. Tyrosine phosphorylation was analysed by immunoblotting with a phosphotyrosine-specific antibody (pTyr). Total amounts of the recombinant proteins were detected with GFP antibody. The figure is representative of two experiments.

DYRK1A and HIPK2 autophosphorylate on a tyrosine residue in the activation loop that is conserved in all members of the HIPK family (Figure 1A-C). To address the question whether all mammalian HIPKs are phosphorylated on tyrosine residues, GFP fusion proteins of HIPK1, HIPK2, HIPK3 and HIPK4 were immunoprecipitated from HeLa cells and tyrosine phosphorylation was assessed by western blot analysis with a phosphotyrosine-specific antibody. GFP-DYRK1A was included as a positive control and GFP served as a negative control. Figure 1D shows that all HIPK members contain immunodetectable phosphotyrosine when isolated from HeLa cells.

To further investigate the mechanism of tyrosine phosphorylation in the HIPK family, we created point mutants of all HIPKs. The crucial tyrosine residue in the activation loop was replaced by phenylalanine to reveal whether this was the only phosphorylatable tyrosine. In addition, kinase-deficient mutants were generated by substituting the catalytic aspartate by asparagine [37]. Guided by our previous analysis of DYRK1A autophosphorylation [34], one series of cells was treated with sodium orthovanadate (Na_3_VO_4_) to inhibit tyrosine phosphatases that might conceal tyrosine kinase activity of mature HIPKs in living cells.

**Figure 2:**
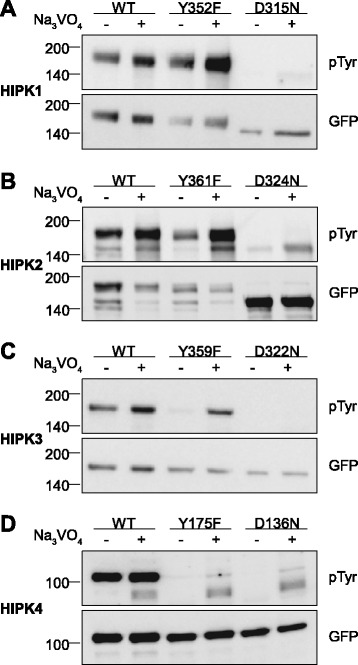
**Tyrosine phosphorylation of HIPK mutants.** HeLa cells were transiently transfected with the indicated expression constructs for HIPK1 **(A)**, HIPK2 **(B)**, HIPK3 **(C)** or HIPK4 **(D)**. Sodium orthovanadate (Na_3_VO_4_) was added to every second sample for 1 h before lysis. GFP fusion proteins were immunoprecipitated and analysed by immunodetection with antibodies for pTyr and GFP. The panels are representative of 2–3 independent experiments.

Firstly, this experiment revealed that the majority of the phosphotyrosine in HIPKs results from autophosphorylation, since phosphotyrosine signals were absent or weak for all kinase-defective HIPK mutants (Figure 2A-D). The residual tyrosine phosphorylation of HIPK2^D324N^ (Figure 2B) may result from either trans-autophosphorylation by endogenous HIPK2 or other tyrosine kinases present in HeLa cells.

Compared to the respective wild-type proteins, the kinase-defective mutants HIPK1^D315N^ and HIPK2^D324N^ showed markedly enhanced electrophoretic mobility (Figure 2A-B). This difference most likely reflects extensive autophosphorylation in the wild type proteins that is absent in the kinase dead mutants. Consistently, wild type HIPK1 and HIPK2 migrated more slowly than predicted from their calculated molecular masses (GFP-HIPK1, 158 kDa, GFP-HIPK2 155 kDa) (Figure 2A-B). Interestingly, the Tyr→Phe mutants migrated with similar electrophoretic mobility as wild type HIPK1 and HIPK2, and vanadate-treatment did not alter migration of HIPK1 or HIPK2. Thus, the observed upshift is attributable to the phosphorylation of serine/threonine rather than tyrosine residues. Indeed, migration of HIPK2 was previously shown to strongly be retarded by treatment with the inhibitor of serine/threonine phosphatases, calyculin A [28].

Secondly, HIPK1^Y352F^ and HIPK2^Y361F^ contained substantial amounts of phosphotyrosine (Figure 2A-B), indicating that these kinases autophosphorylate not only the conserved tyrosine in the activation loop. In agreement with our results, Siepi et al. [29] also detected tyrosine phosphorylation of GFP-HIPK2^Y361F^ and identified several phosphorylated tyrosines by mass spectrometry analysis. In contrast, Saul et al. [28] detected no phosphotyrosine in a FLAG tagged version of this mutant. This difference may be due to the use of different phosphotyrosine-specific antibodies and/or due to the use of different epitope tags. In a direct comparison, we found that the basal phosphotyrosine signal was weaker (but detectable) in FLAG-HIPK2^Y361F^ than in the GFP fusion protein (Additional file 1: Figure S1). Nevertheless, vanadate treatment induced a marked tyrosine autophosphorylation of FLAG-HIPK2^Y361F^.

Treatment with vanadate also revealed that HIPK3^Y359F^ was capable of autophosphorylating tyrosine(s) other than Tyr359, although this reaction was counteracted by tyrosine phosphatases under basal conditions (Figure 2C). In contrast, HIPK4^Y175F^ did not show any phosphotyrosine signal above the background as defined by the catalytically inactive mutant HIPK4^D136N^ (Figure 2D). This difference between HIPK4 and HIPK1-3 may either be due to different enzymatic properties or to the absence of phosphorylatable tyrosines in HIPK4. In particular, HIPK4 lacks the C-terminal tyrosine/histidine-rich domain that harbors 8–10 potentially phosphorylatable tyrosine residues and is conserved in HIPK1-3 (see Figure 1A).

The autophosphorylation on tyrosines outside the catalytic loop suggests that HIPK1-3 may also be able to phosphorylate tyrosines in exogenous substrates. For DYRK1A, which also autophosphorylates on multiple tyrosines [34], no tyrosine site was identified in extensive peptide array analyses [38,39]. Presently, there is no evidence that any member of the HIPK/DYRK families has tyrosine kinase activity towards other proteins.

Vanadate treatment enhanced the tyrosine phosphorylation of HIPK1, HIPK2 and HIPK3 within 1 h, which is very unlikely to reflect solely the newly synthesized protein. To fully exclude this possibility, we performed experiments in cells that were pretreated with the protein synthesis inhibitor cycloheximide. Again, increased phosphotyrosine was detected after vanadate treatment (Additional file 1: Figure S2). This result corroborates the conclusion that tyrosine kinase activity of HIPK1, HIPK2 and HIPK3 is not limited to a transient translational conformation of the catalytic domain. Also of note, the phosphotyrosine signal of HIPK4 was not enhanced by vanadate treatment. This may be due to the lack of tyrosine phosphorylation sites outside the catalytic domain of HIPK4, if the tyrosine in the activation loop is not subject to dephosphorylation by tyrosine phosphatases [30,32]. Similarly, wild type DYRK1A autophosphorylates on tyrosines outside the activation loop, whereas a deletion construct comprising only the catalytic domain does not [34].

### Tyrosine phosphorylation in the activation loop

**Figure 3:**
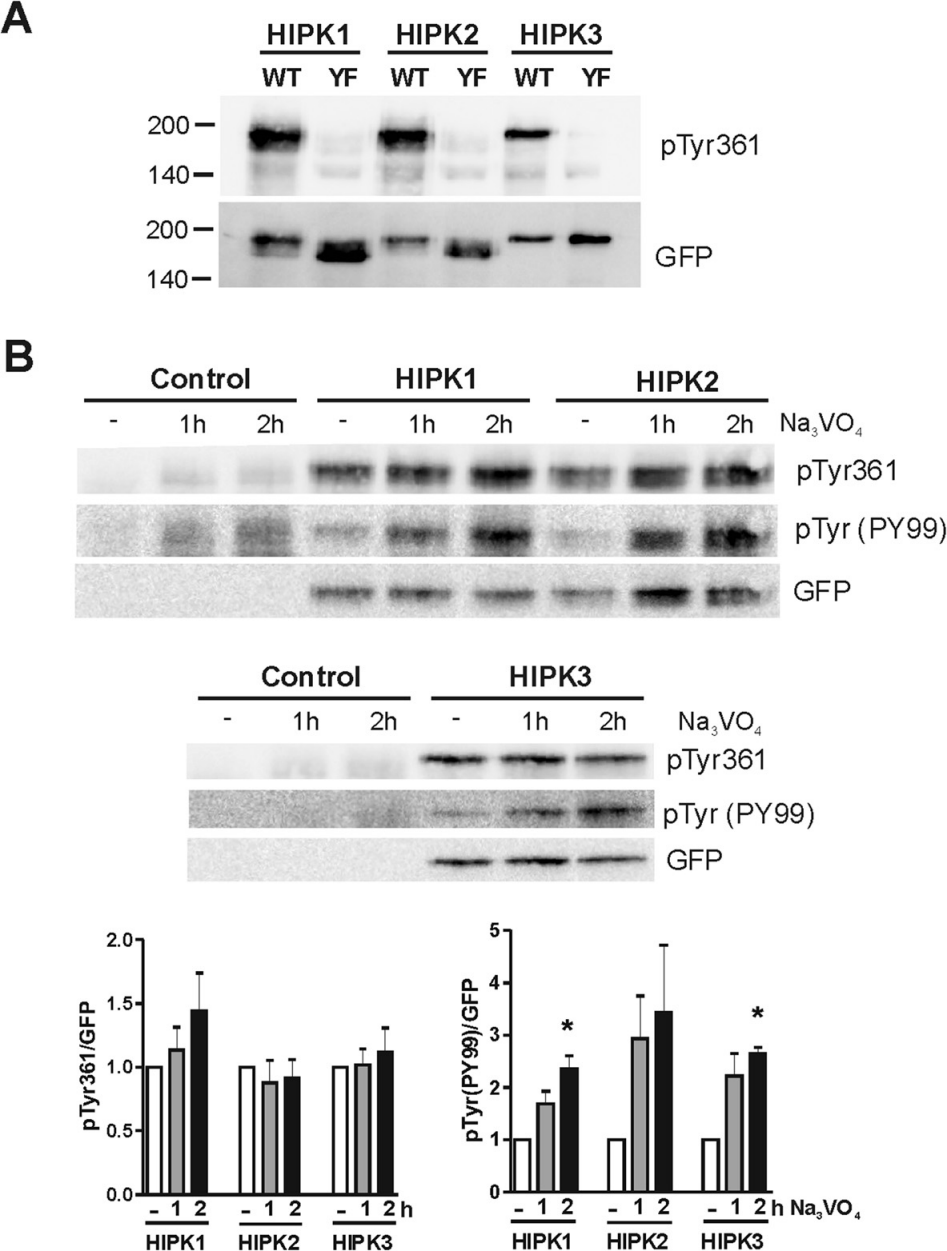
**Tyrosine phosphorylation in the activation loop of HIPK1, HIPK2 and HIPK3.** HeLa cells were transfected with expression plasmids for wild type GFP-HIPK fusion proteins or the Tyr→Phe mutants thereof (YF). If indicated, cells were treated with sodium orthovanadate (Na_3_VO_4_) for 1 h or 2 h. Western blots of total cell lysates were detected with antibodies directed against pTyr361 in HIPK2, a general antibody for phosphotyrosine independent of the sequence context (PY99) and a GFP antibody. **A,** The pTyr361(HIPK2) antibody detects wild type HIPK1-3 but not the Tyr→Phe mutants. **B**, Effect of vanadate treatment. The column diagrams show the quantitative evaluation of 3 experiments. The background signal in the untransfected control samples was subtracted from the signal intensities obtained with the phosphospecific antibodies. Relative phosphorylation after vanadate treatment was calculated by normalization to the signal measured in untreated cells. Means + SEM, * p < 0.05, analysed by one-sample *t* test.

The results shown in Figure 2 were obtained with a general phosphotyrosine antibody and do not allow a specific conclusion on the posttranslational phosphorylation of the activation loop tyrosine. Therefore we took advantage of a new commercial antibody that is directed against phosphorylated Tyr361 in HIPK2. Owing to the sequence similarity of the activation loop (Figure 1C) we expected this antibody to also detect pTyr352 in HIPK1 and pTyr359 in HIPK3. By using the respective Phe→Tyr mutants, we confirmed that the antibody was indeed specific for the activation loop tyrosine of these kinases (Figure 3A). Analysis of HIPK phosphorylation in vanadate-treated cells revealed no significant change in the phosphorylation status of the activation loop tyrosine in HIPK1, HIPK2 and HIPK3 (Figure 3B,C). This result suggests that these phosphotyrosines are not subject to turnover by vanadate-sensitive tyrosine phosphatase activity under these conditions, which argues against a regulatory role of the activation loop tyrosine in the HIPKs. We can however not exclude that changes in the phosphorylation can take place under other circumstances, as reported for HIPK2 [35,36].

### Reduced catalytic activity of the HIPK Tyr→Phe mutants

**Figure 4:**
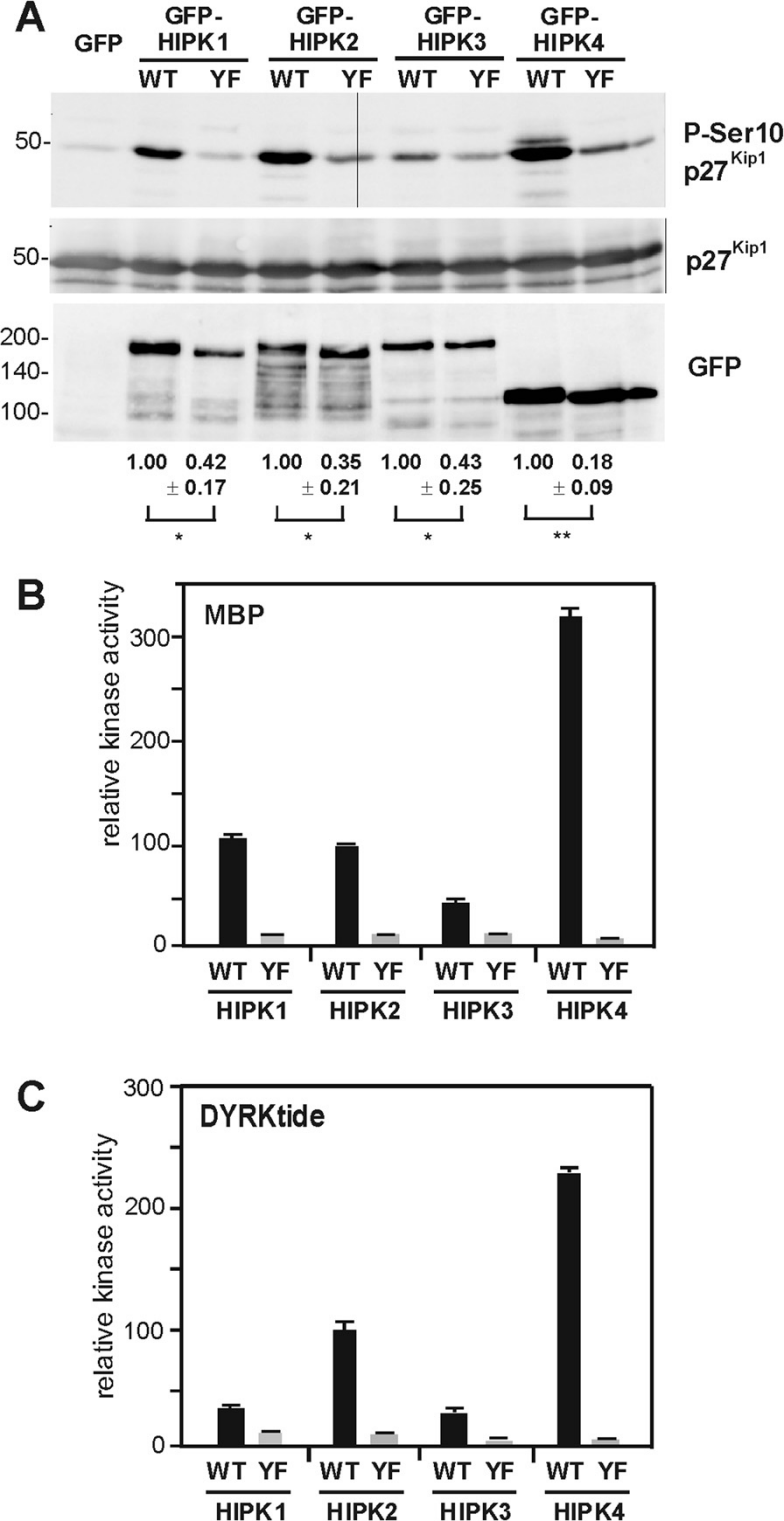
**Maximal activity of HIPKs depends on the activation loop tyrosine.** Wild type GFP-HIPK fusion proteins and the respective Tyr→Phe mutants were immunoprecipitated from HeLa cells and subjected to kinase assays with recombinant GST-p27^Kip1^ **(A)**, myelin basic protein **(B)** or DYRKtide **(C)**. GFP served as background control. **A**, Phosphorylation of p27^Kip1^ at Ser10 was detected by immunoblotting with a phosphorylation-specific antibody. For quantitative evaluation, pSer10 immunoreactivity was normalised to GFP immunoreactivity, which reflects the amount of kinase in the reaction. The blots illustrate a representative experiment, and the relative catalytic activities as determined from 3–4 assays are shown below the panels (means ± SD). One-sample *t* test: *, p < 0.05; **, p < 0.01. **B** and **C**, Phosphorylation of MBP and DYRKtide was measured in triplicate as incorporation of ^32^P. Background values from the GFP control samples were subtracted and activities were normalised to the amount of kinase in the reaction as determined by GFP immunoreactivity. Column diagrams illustrate catalytic activities relative to HIPK2 (WT). The results were replicated in independent experiments, except for a missing value of HIPK1.

The phosphotyrosines in HIPK1^Y352F^, HIPK2^Y361F^ and HIPK3^Y359F^ result from autophosphorylation and thus show that these Tyr→Phe mutants possess catalytic activity. Hence, the catalytic domains of these kinases can adopt an active conformation without tyrosine autophosphorylation in the activation loop. Therefore, we performed *in vitro* kinase assays to assess the Ser/Thr kinase activities of the Tyr→Phe mutants towards exogenous substrates. First, we used recombinant p27^Kip1^ as a substrate, because this CDK inhibitor had been shown to be a substrate of HIPK2 [40]. These assays showed that all HIPKs are capable of phosphorylating Ser10 in p27^Kip1^*in vitro* (Figure 4A). Thus, the sequence similarity of the catalytic domains of HIPK1-4 translates into shared substrate recognition properties. Mutation of the activation loop tyrosine strongly reduced the activity of all HIPKs, supporting the hypothesis that this group of kinases, like the DYRK family, depends on tyrosine autophosphorylation for obtaining full catalytic activity. As tyrosine autophosphorylation of HIPK2 has been shown to control affinity for its substrates [28], we conducted further kinase assays with two other substrates. Myelin basic protein (MBP) was used as an established *in vitro* substrate protein of HIPK2 [7], and we also employed a peptide originally developed as a substrate for DYRK1A [38]. Both substrates were phosphorylated by all HIPKs, as detected by incorporation of ^32^P-labelled phosphate (Figure 4B,C). Wild type HIPK4 was the most active kinase, which may be explained by the absence the C-terminal autoinhibitory domain that is found in the other HIPKs. The Tyr→Phe mutants of all HIPKs showed markedly reduced catalytic towards both substrates. The larger differences between the wild type and mutant enzymes probably reflect the superior properties of the radiometric assay, which provides a more direct and accurate measurement of catalytic activities [41]. Nonetheless, the Tyr→Phe mutants are enzymatically active, consistent with previous reports that HIPK2^Y361F^ has reduced but detectable activity towards different substrates [28,29]. It must be noted that full activity of HIPK2 also depends on the autophosphorylation of a serine residue in the activation loop [28]. This serine in conserved in all HIPKs and DYRKs, and it will be interesting to study the role of this phosphorylation in the regulation of all members of these kinase families.

### Activation loop phosphorylation influences the intracellular localisation of HIPKs

Elimination of the activation loop tyrosine has been reported to alter the subcellular distribution of HIPK2, suggesting that tyrosine autophosphorylation plays a regulatory role in nuclear import or export of HIPK2 [28,29]. To reveal whether this effect was also present in other HIPKs, we compared the subcellular distribution of wild-type and mutant GFP-tagged HIPKs. COS-7 cells were chosen for this analysis because their large, flat cellular shape facilitates microscopic analysis.

**Figure 5:**
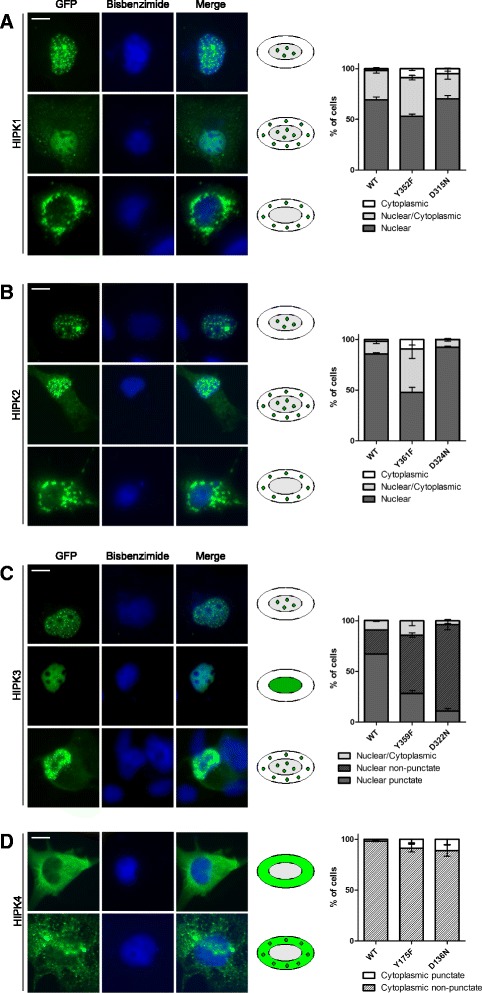
**Subcellular distribution of HIPK mutants.** COS-7 cells were transfected with expression vectors for GFP-HIPK1 **(A)**, GFP-HIPK2 **(B)**, GFP-HIPK3 **(C)** or GFP-HIPK4 **(D)**. The cellular localisation of the HIPK constructs was evaluated by imaging GFP autofluorescence in relation to bisbenzimide-stained nuclei. For each kinase, cells were classified into 3 major patterns that are illustrated by representative images and icons. The cells shown were transfected with (top to bottom) HIPK1 WT, DN, YF; HIPK2 WT, WT, YF; HIPK3 WT, WT, DN and HIPK4 DN, DN. A series of images of the different patterns for each kinase and its mutants is provided in the additional material (Additional file 1: Figure S3). Here the graphs show the percentages of cells classified into the indicated patterns (means ± SD, n = 3). In each of 3 experiments, at least 90 cells were evaluated for all HIPK constructs. Scale bars, 10 μm.

As expected, most COS7 cells expressing wild type HIPK2 showed a dotted distribution in the nucleus, with about 12.5% of the cells also exhibiting cytoplasmic GFP fluorescence (Figure 5B). A significantly larger percentage of HIPK2^Y361F^ expressing cells displayed GFP fluorescence in the cytoplasm, and in some cells HIPK2^Y361F^ was largely excluded from the nucleus (lower panel of Figure 5B, Additional file 1: Figure S3B). This effect of the Tyr→Phe mutation basically reproduces previous observations in U2OS cells reported by Siepi et al. [29]. By co-localisation studies, these authors have tentatively identified the perinuclear structures in which GFP-HIPK2^Y361F^ accumulates as aggresomes. Importantly, however, the majority of cells still contained nuclear HIPK2^Y361F^, clearly indicating that autophosphorylation of Tyr361 is not essential for nuclear import. The localisation of the kinase-deficient mutant HIPK2^D324N^ did not differ from that of wild type HIPK2. This is in contrast to previous observations that the catalytically inactive mutants HIPK2-K221A and K221R lose the localization in nuclear speckles [1,27]. This difference may be due to the use of different point mutants, since the mutation of K221 interferes with the binding of ATP. Such mutants can be functionally distinct from mutants that maintain the integrity of the ATP binding site [42]. The use of different cell lines and levels of overexpression may also have contributed to the observed difference.

As shown in Figure 5A, wild type HIPK1 was also predominantly found in punctate nuclear structures, although the percentage of cells with extranuclear staining was higher than for HIPK2. Interestingly, HIPK1^Y352F^ showed essentially the same pattern of distribution as HIPK2^Y361F^, with about 38% of cells displaying both nuclear and cytoplasmic fluorescence and about 9% nuclear exclusion of HIPK1^Y352F^. Again, the kinase-deficient HIPK1^D315N^ mutant behaved like the wild type protein. Hence, mislocalisation of the HIPK1 and HIPK2 Tyr→Phe mutants is not due to reduced catalytic activity but must be considered a gain-of-function effect (Figure 5A, Additional file 1: Figure S3A). One distinctive feature of the Tyr→Phe mutants of HIPK1 and HIPK2 is their altered residue specificity, with a reduced Ser/Thr kinase activity (Figure 4) and enhanced tyrosine kinase activity (Figure 3). Considering that tyrosine autophosphorylation in the activation loop stabilises the active conformation of the catalytic domain in CMGC kinases [34], the more flexible structure of the Tyr→Phe mutants allows for off-target tyrosine autophosphorylation. These phosphotyrosines may then interfere with protein-protein interactions required for nuclear import.

In spite of the conserved domain structure and the close sequence similarity with HIPK1 and HIPK2, HIPK3 showed a distinct pattern of distribution in COS7 cells (Figure 5C). HIPK3 was almost exclusively detected in the nucleus, and only very few cells with a weak cytoplasmic GFP signal were identified. About one quarter of the cells lacked the typical punctate arrangement of nuclear GFP fluorescence but displayed largely homogeneous nuclear staining. In contrast to HIPK1 and HIPK2, the Y359F mutation of HIPK3 did not cause a significant cytoplasmic redistribution of the kinase (Figure 5C, Additional file 1: Figure S3C). Interestingly, the absence of aggresomes in HIPK3^Y359F^-transfected cells correlates with the observation that this mutant did not contain phosphotyrosine under basal conditions (Figure 3C). However, HIPK3^Y359F^ markedly increased the percentage of cells with homogeneous nuclear fluorescence. This effect was even more pronounced in the catalytically inactive HIPK3^D322N^. Thus, the association of HIPK3 with an as yet undefined subnuclear speckle-like compartment depends on the catalytic activity of the kinase.

In accordance with previous results [4], HIPK4 was excluded from the nucleus and was homogeneously distributed in the cytoplasm in most cells (Figure 5D). Very few cells showed a punctate distribution throughout the cytoplasm. HIPK4^Y175F^ and HIPK4^D136N^ did not significantly differ from the wild type protein in terms of subcellular appearance (Additional file 1: Figure S3D). The different behaviour of HIPK4 compared to HIPK1-3 correlates with the divergent domain structure and the absence of a canonical nuclear localisation signal (Additional file 1: Figure S3E).

## Conclusions

DYRKs have become paradigm examples for their activation mechanism by *cis-*autophosphorylation of a tyrosine residue in the activation-loop. Here we show that all HIPKs also classify as dual specificity kinases that autophosphorylate on tyrosine residues and depend on the activation loop tyrosine for attaining full catalytic activity. The biochemical and structural similarity of the HIPK and DYRK kinase domains supports the hypothesis that activation by tyrosine autophosphorylation is an ancestral feature of CMGC group kinases [30]. Consistently, tyrosine kinase activity is an intrinsic property of the catalytic domain of HIPK2 and does not depend on the cellular context or the presence of the other domains [29,34]. HIPK1-3 also resemble DYRK1A in being capable to autophosphorylate as mature kinases tyrosines other than that in the activation loop. Furthermore, our observation that all HIPKs phosphorylate CDK inhibitor p27^Kip1^ on Ser10 highlights the similarity of target recognition by HIPKs with DYRK1A and DYRK1B [43]. Phosphorylation on Ser10 reduces the proteasomal degradation of p27^Kip1^ and may play a role in the antiapoptotic functions of HIPKs [44].

In contrast to these common properties, HIPKs differ strikingly in the regulatory role of the activation loop tyrosine in subcellular targeting. The increased cytoplasmic localisation of HIPK1^Y352F^ and HIPK2^Y361F^ was not found in the respective mutants of HIPK3 (Figure 5C) or DYRK1A [29]. Interestingly, overexpression of the oncogenic tyrosine kinase Src has recently been reported to enhance tyrosine phosphorylation of HIPK2 and redistribute the kinase to the cytoplasm [36]. Further studies will be necessary to understand how tyrosine phosphorylation affects the tumor-suppressive function of HIPK1 and HIPK2 in human cancers and to reveal the physiological or pathophysiological relevance of tyrosine autophosphorylation in the regulation of the other member of the HIPK family.

## Material and methods

### Antibodies

The following antibodies were used: mouse monoclonal antibodies against phosphotyrosine (PY99, sc-7020, Santa Cruz Biotechnology, Santa Cruz, CA, USA) and p27^Kip1^ (BD Transduction laboratories, Heidelberg, Germany), rabbit monoclonal antibodies specific for pS10-p27^Kip1^ (#ab62364, Abcam, Cambridge, UK) or pTyr361-HIPK2 (#PA5-13045, Thermo Scientific, Rockford, IL, USA) and polyclonal goat antibody against green fluorescent protein (GFP, Rockland Immunochemicals Inc., 600-101-215, Gilbertsville, PA, USA). A rat monoclonal HIPK2 antibody was kindly provided by M. Lienhardt Schmitz (Justus-Liebig-University, Giessen, Germany).

### Plasmids

The vector plasmid pEGFP-C1 (Clontech Laboratories, Mountain View, CA) was used for mammalian expression of HIPK fusion proteins with an N-terminal GFP tag. Expression plasmids for GFP-HIPK1, GFP-HIPK2 and FLAG-HIPK2 were kindly provided by Cheol Yong Choi (Sungkyunkwan University, Suwon, Korea) and M. Lienhardt Schmitz (Table 1). The HIPK2 clones differ from the human reference sequence in the Uniprot database in two amino acid residues (H233R, F378S). Rat HIPK3 cDNA was cut from pFLAG-CMV2-rHIPK3 (gift of Jorma Palvimo, University of Eastern Finland, Kuopio, Finland) with *Eco*RI and subcloned into pEGFP-C1. This cDNA differed by one mismatch (A963V) from the Uniprot reference sequence. HIPK4 was PCR amplified using a human cDNA clone (MGC:26964, Openbiosystems, Huntsville, AL) as template and ligated into pEGFP-C1 using N-terminal *Bgl*II and C-terminal *Eco*RI sites. Correctness of PCR and cloning was verified by DNA sequencing. Point mutants were created using the QuikChange Site-Directed Mutagenesis Kit (Stratagene, La Jolla, CA, USA) and verified by DNA sequencing. The designation of point mutants refers to the numbering of the reference proteins listed in Table 1 that are defined as canonical sequence variants in the Uniprot database (www.uniprot.org). Since two possible start codons exist in HIPK2, the activation loop tyrosine in HIPK2 has been numbered as Tyr354 in other reports [28,29].

**Table 1:** **List of GFP-HIPK expression clones**

**GFP fusion protein**	**NCBI reference sequence**	**Canonical uniprot sequence**	**Reference**
GFP-mHIPK1	NM_010432.2	O88904	[26]
GFP-hHIPK2	NM_001113239	Q9H2X6	[8]
GFP-rHIPK3	NM_031787	O88850	[22]
GFP-hHIPK4	NM_144685	Q8NE63	--

### Cell culture and immunoprecipitation of GFP fusion proteins

HeLa cells were cultivated on 100-mm plates in DMEM (Dulbecco’s modified eagle medium) containing 10% FCS (fetal calf serum) at 37°C and 5% CO_2._ Cells were transfected with expression vectors for HIPK fusion proteins using FuGENE HD (Promega, Mannheim, Germany). If indicated, sodium orthovanadate (Na_3_VO_4_) was added to the HeLa medium to a final concentration of 100 μM 1 h before cell lysis. After 48 h, cells were washed and lysed in 1 ml of native lysis buffer (50 mM TrisHCl pH 7.5, 150 mM NaCl, 2 mM EDTA, 0.5% NP-40, supplemented with 1 mM Na_3_VO_4_, 1 mM phenylmethylsulfonylfluoride and 10 μg/ml each of aprotinin, pepstatin and leupeptin). Samples were cleared off cell debris by centrifugation and incubated with 15 μl GFP-Trap® (ChromoTek, Martinsried, Germany) for 1 h at 4°C in and end-over-end rotator. GFP-Trap® beads were collected using a magnetic rack and washed three times with washing buffer (50 mM TrisHCl pH 7.5, 150 mM NaCl, 2 mM EDTA, 0.1% NP-40). Bound proteins were eluted in 10 μl Laemmli Sample Buffer supplemented with 6 μg/100 μl dithiothreitol at 95°C and resolved by SDS-PAGE.

### Immunocomplex kinase assays

To measure the relative catalytic activities of the HIPK Tyr→Phe mutants, GFP-Trap® immunoprecipitates were equilibrated with kinase buffer (25 mM Hepes pH 7.0, 5 mM MgCl_2_, 0.5 mM dithiothreitol). Non-radioactive assays were performed in a total volume of 20 μL for 30 min at 30°C in the presence of 500 μM ATP and 20 ng/μL of GST-p27, a fusion protein of glutathione S-transferase and human p27^Kip1^. GST-p27 was produced in *Escherichia coli* from the plasmid pGEX-5x-3-p27^Kip1^ (kindly provided by J. Vervoorts-Weber, Institute of Biochemistry, RWTH Aachen University, Germany) and purified by affinity adsorption to glutathione Sepharose. Phosphorylation of Ser10 was detected by western blot analysis, and relative catalytic activity was calculated by normalising the p27(pSer10) signal to the amount of kinase in the reaction, as determined by the intensity of the GFP signal. Radiometric assays were performed in a volume of 30 μL for 15 min at 30°C in the presence of 50 μM myelin basic protein from bovine brain (Sigma) or 200 μM DYRKtide [39] and 10 μM of [γ-^32^P]ATP. Phosphate incorporation was determined by the phosphocellulose method. Each data point was determined in triplicate, and the linearity of the reactions was verified by measuring incorporation of ^32^P after 30 min. Activities of wild type HIPK4 exceeded the linear range of the assay and may be underestimated.

### Western blotting

Samples were separated by SDS-PAGE using 8% acrylamide gels and blotted onto nitrocellulose membranes. Membranes were blocked with 3% BSA in TBS-T buffer (20 mM Tris–HCl pH 7.6, 137 mM NaCl, 0.2% Tween-20) and incubated with the primary antibodies in a 1:1000 dilution at 4°C overnight. After washing, membranes were incubated with secondary antibodies for 1 h. Chemiluminescence signals were detected with a LAS-3000 CCD imaging system and densitometrically quantitated using the AIDA image analysis software (Raytest, Straubenhardt, Germany).

### Fluorescence microscopy

Glass coverslips (15 mm diameter) were transferred into 6-well plates and wells were filled with cell culture medium. Subsequently, COS-7 cells were seeded and grown for 24 h at 37°C in DMEM containing 10% FCS at 5% CO_2_ before transfection with GFP-HIPK expression vectors using FuGENE HD. After incubation for additional 48 h, cells were carefully washed once with PBS buffer and fixed using 1 ml 4% paraformaldehyde in PBS buffer for 10 minutes. Cells were then washed three times in 50 mM Tris-HCl pH 7.6 and incubated with bisbenzimide (1:4000 dilution in 50 mM TrisHCl pH 7.6) for 10 minutes in darkness in order to stain nuclear DNA. After that, coverslips were mounted onto object slides using Immu-Mount™ (Thermo Fisher Scientific, Waltham, MA). Cells were then analysed using a Axiovert 200 M inverted microscope (Zeiss, Jena, Germany). Only cells with sufficient GFP and bisbenzimide signal as well as vital appearance were counted, aiming to count 100 cells per slide. Due to inadequate transfection or fixation rates only >90 cells could be evaluated in 2 cases. Images were processed using Zeiss AxioVision 4.7 software. One-way ANOVA and Tukey’s Multiple Comparison Test were applied to test significant differences between mutants and wild type using the GraphPad Prism 5 software (GraphPad Software Inc, La Jolla, CA, USA).

## Additional file

Additional file 1:
**Figure S1.** Tyrosine autophosphorylation in different FLAG-HIPK2 and GFP-HIPK2, HIPK2^Y361F^. **Figure S2**. Tyrosine autophosphorylation of GFP-HIPK1-3 in the presence of cycloheximide. **Figures S3** and **S4** contain additional images which illustrate the different patterns of subcellular distribution for all HIPK constructs (supporting information to Figure 5).

## References

[1] Kim YH, Choi CY, Kim Y (1999). Covalent modification of the homeodomain-interacting protein kinase 2 (HIPK2) by the ubiquitin-like protein SUMO-1. Proc Natl Acad Sci U S A.

[2] Schmitz ML, Rodriguez-Gil A, Hornung J (2014). Integration of stress signals by homeodomain interacting protein kinases. Biol Chem.

[3] Manning G, Whyte DB, Martinez R, Hunter T, Sudarsanam S (2002). The protein kinase complement of the human genome. Science.

[4] Arai S, Matsushita A, Du K, Yagi K, Okazaki Y, Kurokawa R (2007). Novel homeodomain-interacting protein kinase family member, HIPK4, phosphorylates human p53 at serine 9. FEBS Lett.

[5] Rinaldo C, Prodosmo A, Siepi F, Soddu S (2007). HIPK2: a multitalented partner for transcription factors in DNA damage response and development. Biochem Cell Biol.

[6] Calzado MA, Renner F, Roscic A, Schmitz ML (2007). HIPK2: a versatile switchboard regulating the transcription machinery and cell death. Cell Cycle.

[7] D’Orazi G, Cecchinelli B, Bruno T, Manni I, Higashimoto Y, Saito S (2002). Homeodomain-interacting protein kinase-2 phosphorylates p53 at Ser 46 and mediates apoptosis. Nat Cell Biol.

[8] Hofmann TG, Möller A, Sirma H, Zentgraf H, Taya Y, Dröge W (2002). Regulation of p53 activity by its interaction with homeodomain-interacting protein kinase-2. Nat Cell Biol.

[9] Möller A, Sirma H, Hofmann TG, Rueffer S, Klimczak E, Dröge W (2003). PML is required for homeodomain-interacting protein kinase 2 (HIPK2)-mediated p53 phosphorylation and cell cycle arrest but is dispensable for the formation of HIPK domains. Cancer Res.

[10] Puca R, Nardinocchi L, Givol D, D’Orazi G (2010). Regulation of p53 activity by HIPK2: molecular mechanisms and therapeutical implications in human cancer cells. Oncogene.

[11] Sombroek D, Hofmann TG (2009). How cells switch HIPK2 on and off. Cell Death Differ.

[12] Saul VV, Schmitz ML (2013). Posttranslational modifications regulate HIPK2, a driver of proliferative diseases. J Mol Med.

[13] Rinaldo C, Siepi F, Prodosmo A, Soddu S (2008). HIPKs: Jack of all trades in basic nuclear activities. Biochim Biophys Acta.

[14] Moehlenbrink J, Bitomsky N, Hofmann TG (2010). Hypoxia suppresses chemotherapeutic drug-induced p53 Serine 46 phosphorylation by triggering HIPK2 degradation. Cancer Lett.

[15] Hofmann TG, Glas C, Bitomsky N (2013). HIPK2: a tumour suppressor that controls DNA damage-induced cell fate and cytokinesis. Bioessays.

[16] Aikawa Y, Nguyen LA, Isono K, Takakura N, Tagata Y, Schmitz ML (2006). Roles of HIPK1 and HIPK2 in AML1- and p300-dependent transcription, hematopoiesis and blood vessel formation. EMBO J.

[17] Kondo S, Lu Y, Debbas M, Lin AW, Sarosi I, Itie A (2003). Characterization of cells and gene-targeted mice deficient for the p53-binding kinase homeodomain-interacting protein kinase 1 (HIPK1). Proc Natl Acad Sci U S A.

[18] Wiggins AK, Wei G, Doxakis E, Wong C, Tang AA, Zang K (2004). Interaction of Brn3a and HIPK2 mediates transcriptional repression of sensory neuron survival. J Cell Biol.

[19] Isono K, Nemoto K, Li Y, Takada Y, Suzuki R, Katsuki M (2006). Overlapping roles for homeodomain-interacting protein kinases hipk1 and hipk2 in the mediation of cell growth in response to morphogenetic and genotoxic signals. Mol Cell Biol.

[20] Inoue T, Kagawa T, Inoue-Mochita M, Isono K, Ohtsu N, Nobuhisa I (2010). Involvement of the Hipk family in regulation of eyeball size, lens formation and retinal morphogenesis. FEBS Lett.

[21] Ecsedy JA, Michaelson JS, Leder P (2003). Homeodomain-interacting protein kinase 1 modulates Daxx localization, phosphorylation, and transcriptional activity. Mol Cell Biol.

[22] Moilanen AM, Karvonen U, Poukka H, Jänne OA, Palvimo JJ (1998). Activation of androgen receptor function by a novel nuclear protein kinase. Mol Biol Cell.

[23] Sierra OL, Towler DA (2010). Runx2 trans-activation mediated by the Msx2-interacting nuclear target requires homeodomain interacting protein kinase-3. Mol Endocrinol.

[24] Shojima N, Hara K, Fujita H, Horikoshi M, Takahashi N, Takamoto I (2012). Depletion of homeodomain-interacting protein kinase 3 impairs insulin secretion and glucose tolerance in mice. Diabetologia.

[25] da Locke JM, Silva Xavier G, Dawe HR, Rutter GA, Harries LW (2014). Increased expression of miR-187 in human islets from individuals with type 2 diabetes is associated with reduced glucose-stimulated insulin secretion. Diabetologia.

[26] Kim YH, Choi CY, Lee SJ, Conti MA, Kim Y (1998). Homeodomain-interacting protein kinases, a novel family of co-repressors for homeodomain transcription factors. J Biol Chem.

[27] De la Vega L, Fröbius K, Moreno R, Calzado MA, Geng H, Schmitz ML (1813). Control of nuclear HIPK2 localization and function by a SUMO interaction motif. Biochim Biophys Acta.

[28] Saul VV, de la Vega L, Milanovic M, Krüger M, Braun T, Fritz-Wolf K (2013). HIPK2 kinase activity depends on cis-autophosphorylation of its activation loop. J Mol Cell Biol.

[29] Siepi F, Gatti V, Camerini S, Crescenzi M, Soddu S (1833). HIPK2 catalytic activity and subcellular localization are regulated by activation-loop Y354 autophosphorylation. Biochim Biophys Acta.

[30] Becker W, Sippl W (2011). Activation, regulation, and inhibition of DYRK1A. FEBS J.

[31] Himpel S, Panzer P, Eirmbter K, Czajkowska H, Sayed M, Packman LC (2001). Identification of the autophosphorylation sites and characterization of their effects in the protein kinase DYRK1A. Biochem J.

[32] Lochhead PA, Sibbet G, Morrice N, Cleghon V (2005). Activation-loop autophosphorylation is mediated by a novel transitional intermediate form of DYRKs. Cell.

[33] Lochhead PA, Kinstrie R, Sibbet G, Rawjee T, Morrice N, Cleghon V (2006). A chaperone-dependent GSK3beta transitional intermediate mediates activation-loop autophosphorylation. Mol Cell.

[34] Walte A, Rüben K, Birner-Gruenberger R, Preisinger C, Bamberg-Lemper S, Hilz N (2013). Mechanism of dual specificy kinase activity of DYRK1A. FEBS J.

[35] Shang Y, Doan CN, Arnold TD, Lee S, Tang AA, Reichardt LF (2013). Transcriptional corepressors HIPK1 and HIPK2 control angiogenesis via TGF-β-TAK1-dependent mechanism. PLoS Biol.

[36] Polonio-Vallon T, Kirkpatrick J, Krijgsveld J, Hofmann TG (2014). Src kinase modulates the apoptotic p53 pathway by altering HIPK2 localization. Cell Cycle.

[37] Hanks SK, Hunter T (1995). Protein kinases 6. The eukaryotic protein kinase superfamily: kinase (catalytic) domain structure and classification. FASEB J.

[38] Himpel S, Tegge W, Frank R, Leder S, Joost HG, Becker W (2000). Specificity determinants of substrate recognition by the protein kinase DYRK1A. J Biol Chem.

[39] Papadopoulos C, Arato K, Lilienthal E, Zerweck J, Schutkowski M, Chatain N (2011). Splice variants of the dual specificity tyrosine phosphorylation-regulated kinase 4 (DYRK4) differ in their subcellular localization and catalytic activity. J Biol Chem.

[40] Pierantoni GM, Esposito F, Tornincasa M, Rinaldo C, Viglietto G, Soddu S (2011). Homeodomain-interacting protein kinase-2 stabilizes p27(kip1) by its phosphorylation at serine 10 and contributes to cell motility. J Biol Chem.

[41] Hastie CJ, McLauchlan HJ, Cohen P (2006). Assay of protein kinases using radiolabeled ATP: a protocol. Nat Protoc.

[42] Cameron AJ, Escribano C, Saurin AT, Kostelecky B, Parker PJ (2009). PKC maturation is promoted by nucleotide pocket occupation independently of intrinsic kinase activity. Nat Struct Mol Biol.

[43] Soppa U, Schumacher J, Florencio Ortiz V, Pasqualon T, Tejedor FJ, Becker W (2014). The Down syndrome-related protein kinase DYRK1A phosphorylates p27Kip1 and Cyclin D1 and induces cell cycle exit and neuronal differentiation. Cell Cycle.

[44] Besson A, Gurian-West M, Chen X, Kelly-Spratt KS, Kemp CJ, Roberts JM (2006). A pathway in quiescent cells that controls p27Kip1 stability, subcellular localization, and tumor suppression. Genes Dev.

