# Supplementary material for: Effect of tyrosine autophosphorylation on catalytic activity and subcellular localisation of homeodomain-interacting protein kinases (HIPK)
Source: Cell Commun Signal. 2015 Jan 29;13:3. doi: 10.1186/s12964-014-0082-6 (PMC4353451; doi:10.1186/s12964-014-0082-6)
Supplement: Additional file 1: — Figure S1. Tyrosine autophosphorylation in different FLAG-HIPK2 and GFP-HIPK2, HIPK2Y361F. Figure S2. Tyrosine autophosphorylation of GFP-HIPK1-3 in the presence of cycloheximide. Figures S3 and S4 contain additional images which illustrate the different patterns of subcellular distribution for all HIPK constructs (supporting information to Figure 5). [file 12964_2014_82_MOESM1_ESM.pdf]

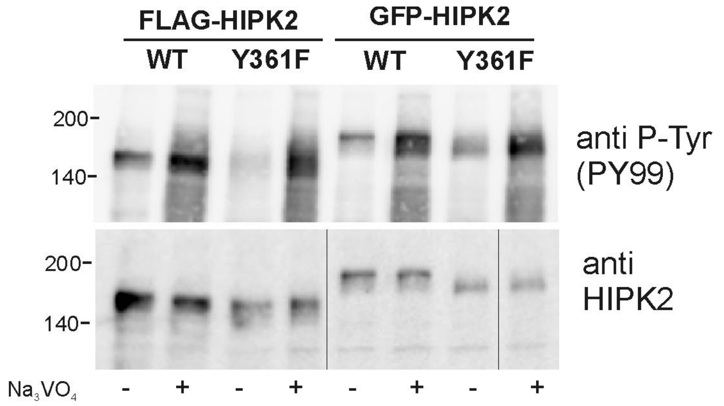


**Figure S1 – Tyrosine autophosphorylation of HIPK2 wt and HIPK2$^{Y361F}$**
HeLa cells expressing either FLAG-HIPK2 or GFP-HIPK2, each wild type and the Y361F mutant, were treated with sodium orthovanadate for 1 h before lysis or left untreated. Total cellular lysates were analysed by immunodetection with antibodies for pTyr (PY99 antibody) and HIPK2.

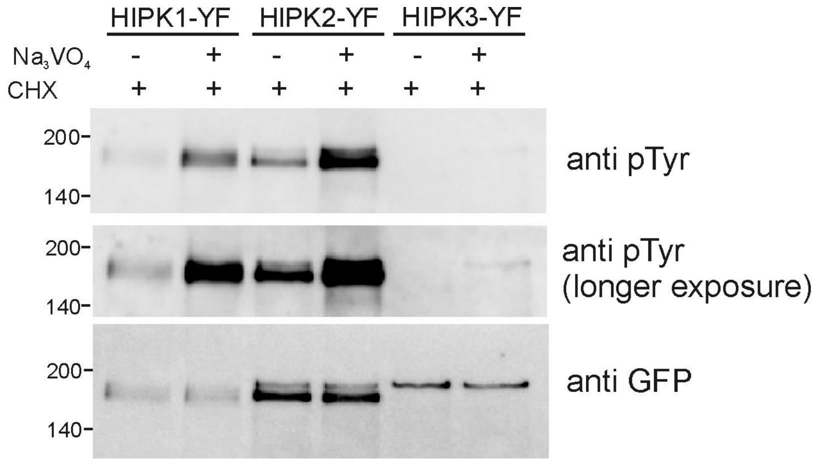


**Figure S2 – Tyrosine autophosphorylation of GFP-HIPK1-3 in the presence of cycloheximid (CHX)**
HeLa cells expressing the GFP-HIPK1-3 Tyr→Phe mutants were treated with CHX (10µg/ml). After 10 min, sodium orthovanadate (Na3VO4) was added to every second sample for 1 h before lysis. GFP fusion proteins were immunoprecipitated and analysed by immunodetection with antibodies for pTyr and GFP.

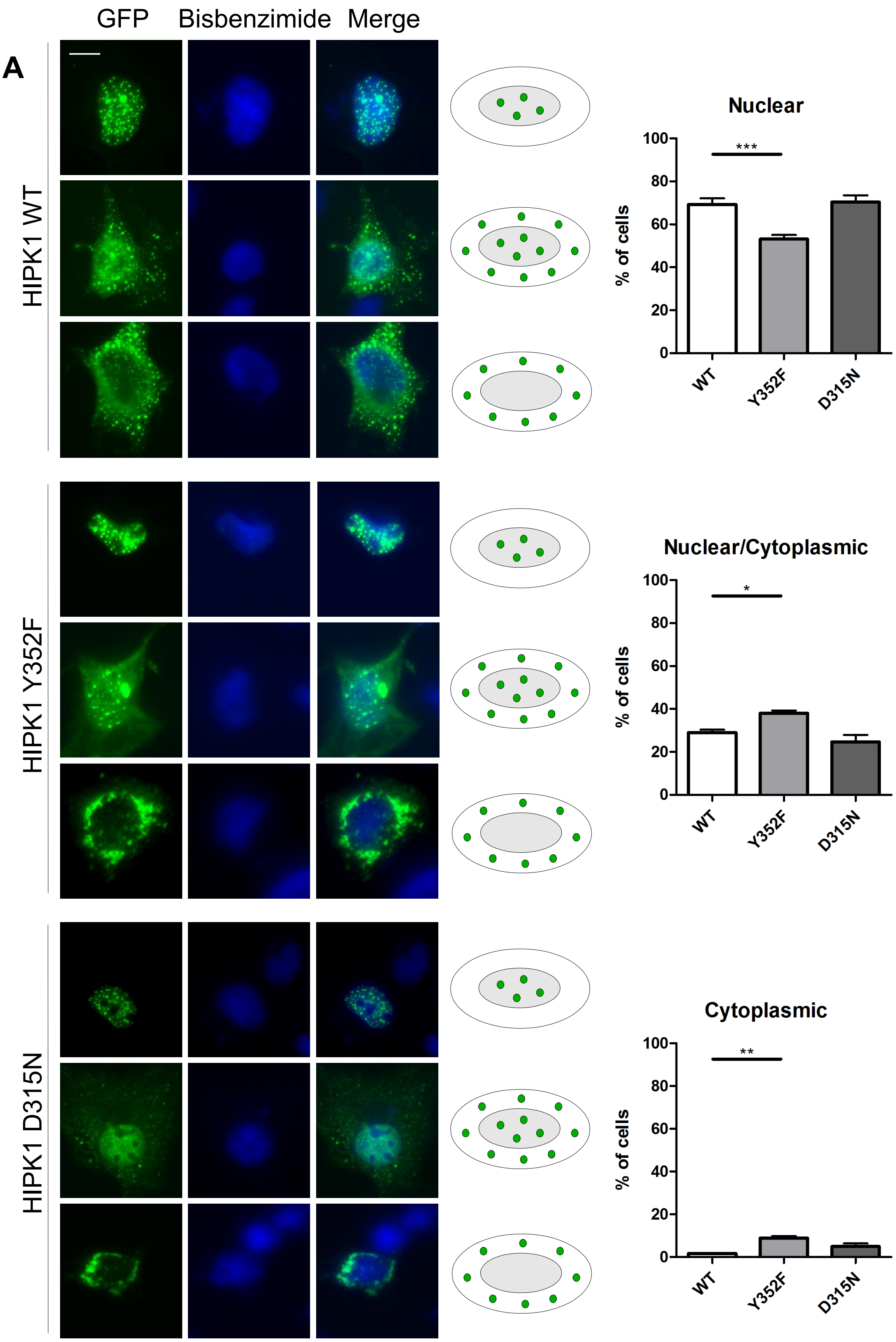
GFP
Bisbenzimide
Merge
A
HIPK1 WT
HIPK1 Y352F
HIPK1 D315N
Nuclear
Nuclear/Cytoplasmic
Cytoplasmic
% of cells
100
80
60
40
20
0
***
*
**
WT
Y352F
D315N

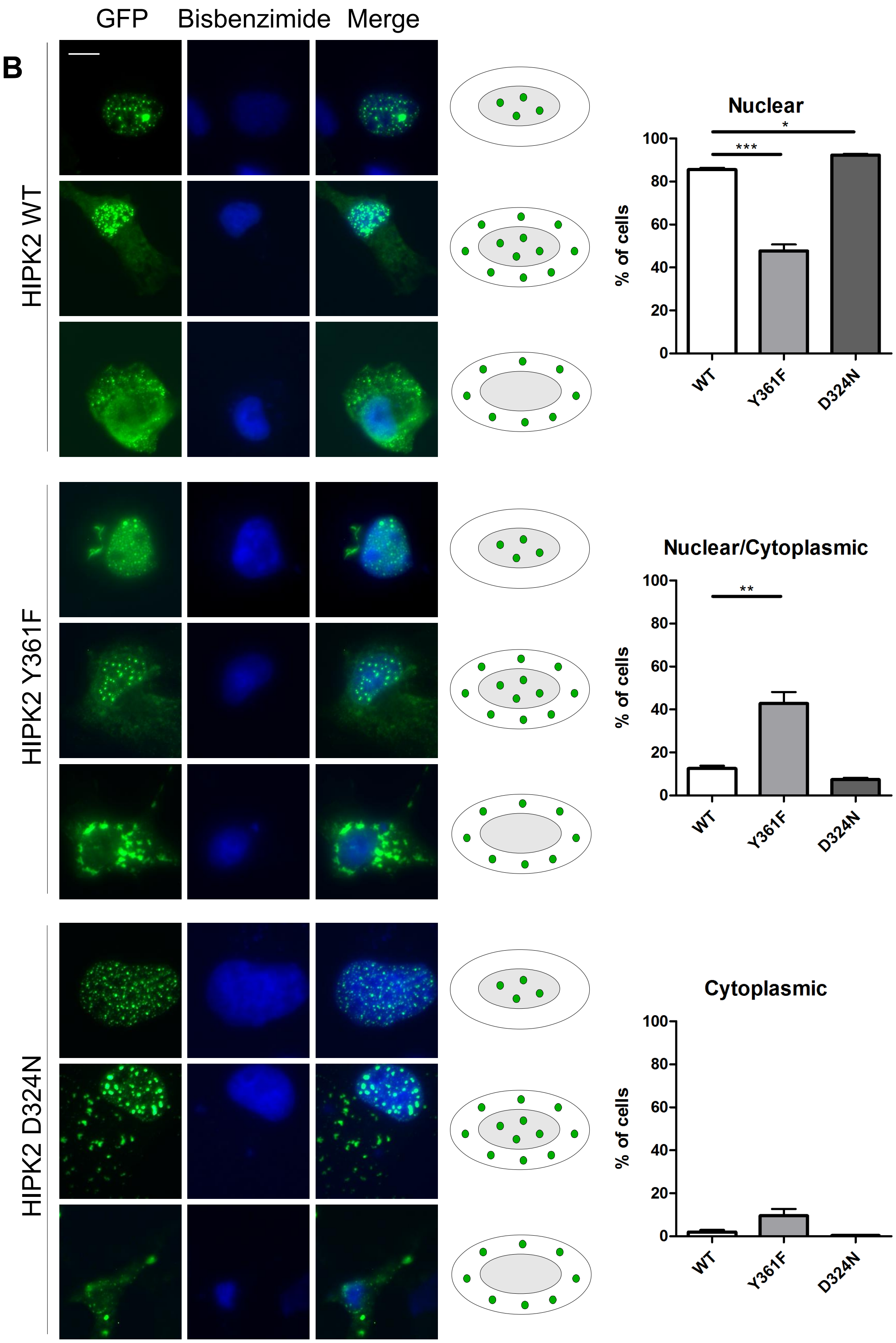
GFP
Bisbenzimide
Merge
B
HIPK2 WT
HIPK2 Y361F
HIPK2 D324N
Nuclear
Nuclear/Cytoplasmic
Cytoplasmic
% of cells
100
80
60
40
20
0
***
*
**
WT
Y361F
D324N

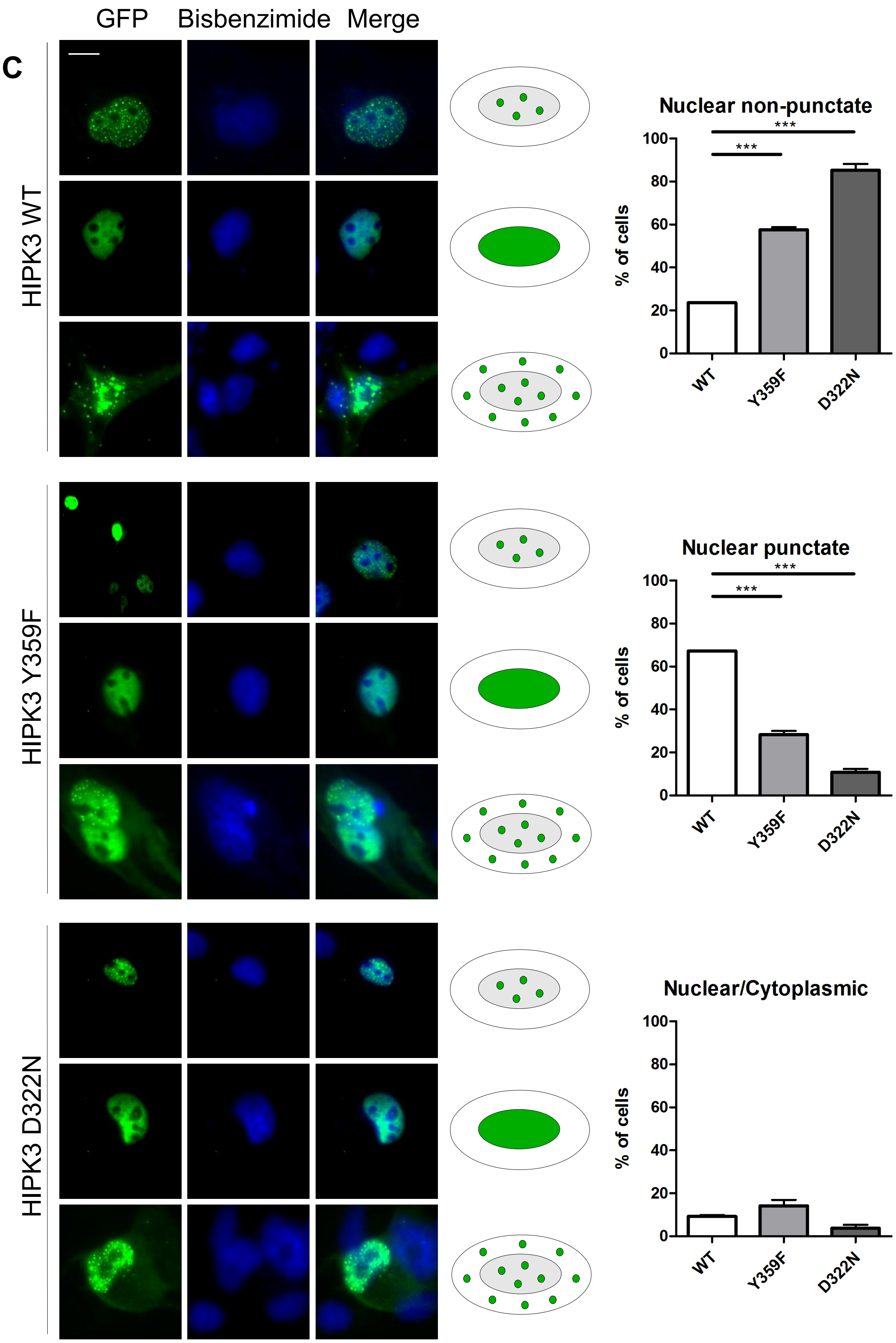
GFP
Bisbenzimide
Merge
C
HIPK3 WT
HIPK3 Y359F
HIPK3 D322N
Nuclear non-punctate
Nuclear punctate
Nuclear/Cytoplasmic
% of cells
***
100
80
60
40
20
0
WT
Y359F
D322N

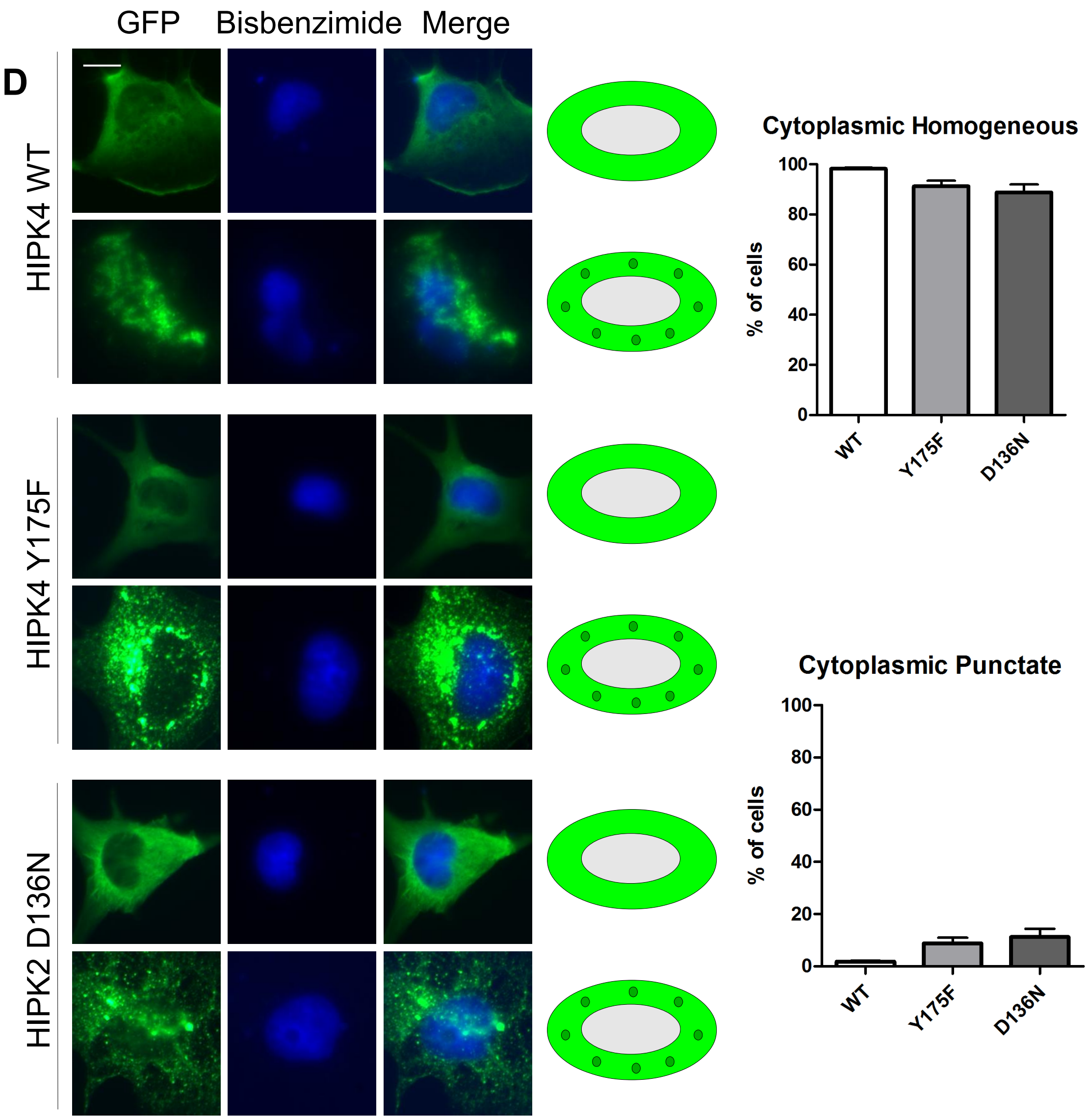


**E**

| | NLS1 | NLS2 | NLS3 |
|---|---|---|---|
| HIPK1 | 122 GLKRKSEE | 839 SSLPSKKNKQSA | 866 -------------YSLV |
| HIPK2 | 130 GLKRKSEE | 797 STTSSRKSKQHQ | 830 RS-KRVKENTPPRCAMV |
| HIPK3 | 126 GLKRKSEE | 758 TTKKNKLCQNRS | 787 ISGKEVEE---VSCVET |

**Figure S3 – Subcellular localisation of GFP-HIPK1-4 and point mutations thereof**
The images are representative for the different patterns of subcellular distribution for all HIPK constructs (supporting information to Fig. 4). The corresponding graphs show percentages of each pattern. The point mutants were compared to the wild type kinases via one-way ANOVA and Tukey's multiple comparison test (n=3): *, $p < 0.05$; **, $p < 0.01$; ***, $p < 0.005$.
The following panels were selected to illustrate the different patterns of distribution in Fig. 4:
**(A)** nuclear, HIPK1 WT; nuclear/cytoplasmic, HIPK1 D315N; cytoplasmic, HIPK1 Y354F; **(B)** nuclear and nuclear/cytoplasmic, HIPK2 WT; cytoplasmic, HIPK2 Y361F; **(C)** nuclear punctate and nuclear non-punctate, HIPK3 WT; nuclear/cytoplasmic, HIPK3 D322N; **(D)** cytoplasmic punctate and cytoplasmic non-punctate, HIPK4 D136N; **(E)** Nuclear localisation signals in HIPK1, HIPK2 and HIPK3.

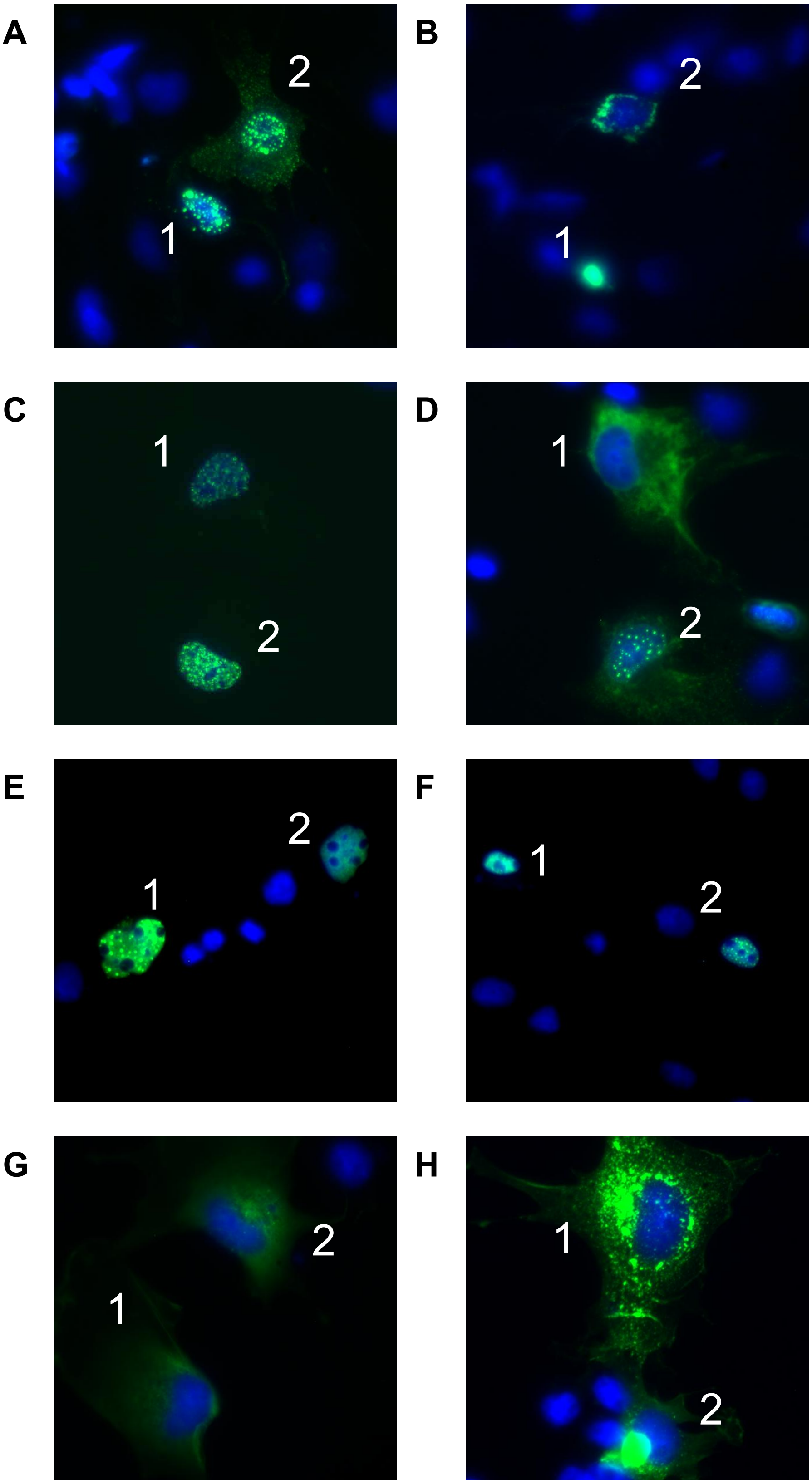


**Figure S4 – Coexisting localisation patterns of GFP-HIPK1-4 and point mutations**
The images representatively show different HIPK mutants coexisting in different distribution patterns. **(A)** HIPK1 wt, nuclear (1), nuclear/cytoplasmic (2); **(B)** HIPK1 D315N, nuclear (1), cytoplasmic (2); **(C)** HIPK2 wt, nuclear (1, 2); **(D)** HIPK2 Y361F, cytoplasmic (1), nuclear/cytoplasmic (2); **(E)** HIPK3 wt, nuclear punctate (1), nuclear non-punctate (2); **(F)** HIPK3 D322N, nuclear non-punctate (1), nuclear punctate (2); **(G)** HIPK4 Y175F, cytoplasmic punctate (1, 2); **(H)** HIPK4 Y175F, cytoplasmic punctate (1), cytoplasmic non-punctate (2).
